# The Female Impact in the World of Neurodegeneration

**DOI:** 10.3389/fnint.2021.750603

**Published:** 2021-10-06

**Authors:** Celeste Gonzalez Osorio, Pragnya Guduru, Nico Osier

**Affiliations:** ^1^College of Liberal Arts, University of Texas at Austin, Austin, TX, United States; ^2^College of Natural Sciences, University of Texas at Austin, Austin, TX, United States; ^3^University of Texas at Austin School of Nursing, Austin, TX, United States; ^4^Department of Neurology Austin, Dell Medical School, Austin, TX, United States

**Keywords:** women, neurodegeneration, tau, Alzheimer's disease, alpha-synuclein, dementia, neuroscience, Huntington's disease

## Introduction

Scientific research fuels advances in medicine, but the contributions of individual scientists, especially women, are often underappreciated. The field of neuroscience is vast, with many important gaps in knowledge remaining; thus, it is imperative for women to be able to contribute. A pivotal 2018 study revealed that women were still shockingly underrepresented in academic neurology, representing only 30.8% of academic neurologists (McDermott et al., 2018). Of additional concern, this gender gap grows with increasing academic rank, with only 13.8% of neurology professors being women (McDermott et al., 2018). Although women have faced much adversity in the field of neuroscience, there have been pioneers who have paved a path for other women. Moreover, these women made important contributions that enhanced scientific understanding of neurodegeneration and neurodegenerative diseases. The purpose of this paper is to highlight notable, but underappreciated, female neuroscientists and their important contributions to modern understanding of neurodegeneration.

### Overview of Neurodegeneration

The term neurodegeneration is a mixture of two words—“neuro”, which refers to nerve cells and then “degeneration”, which refers to progressive damage. The term in its entirety, “neurodegeneration”, can be used to describe various conditions that result in the loss of nerve structure and function. This deterioration of neural structures results in a loss of cognitive abilities such as memory and decision making (Murman, 2015). Loss of neural function is a key hallmark in many neurodegenerative diseases, including Parkinson's disease (PD), Huntington's disease (HD), Multiple Sclerosis (MS), Alzheimer's disease (AD), and many rare disorders (Przedborski et al., 2003).

**Figure 1:**
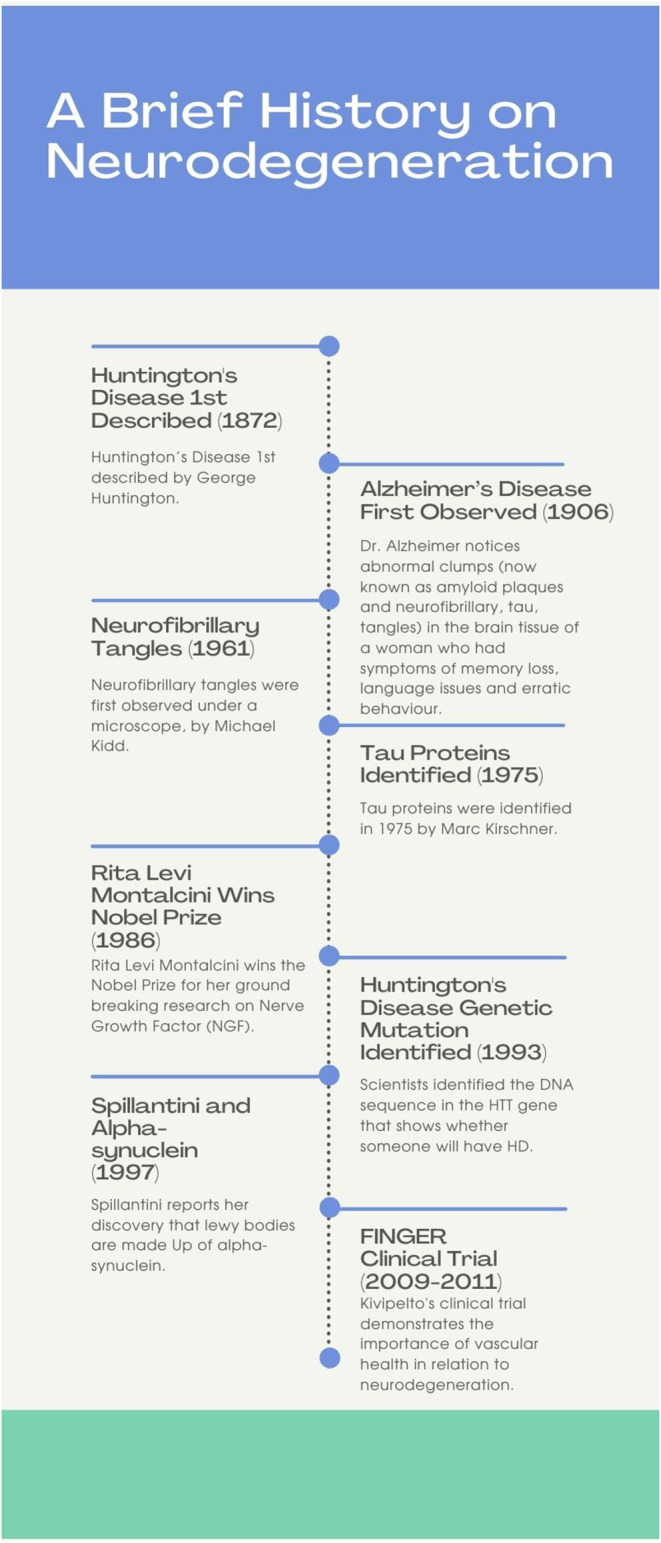
The above timeline outlines a few key events that contributed to knowledge of neurodegeneration. Some of the events shown are the first descriptions of neurodegenerative diseases, others are key discoveries regarding the mechanisms of neurodegeneration, one is a clinical trial.

An important area of research is exploring the mechanisms that underlie neurodegeneration and plasticity; this includes investigation of proteins implicated in AD (e.g., tau; amyloid-beta) and Parkinson's (e.g., alpha-synuclein). Tau is a protein found in the brain that serves to stabilize microtubules (Gao et al., 2018); tau hyperphosphorylation results in its aggregation into insoluble tangles, a characteristic biomarker of AD. Another characteristic feature of AD is the aggregation of amyloid beta protein into extracellular deposits known as amyloid plaques (Bloom, 2014). Alpha-synuclein is a neuronal protein that normally regulates trafficking of synaptic vesicles for neurotransmitter release. If mutated, the once soluble protein aggregates into insoluble fibrils forming Lewy bodies, a hallmark feature of PD (Stefanis, 2012). Notably, there are currently no known therapies that can prevent or reverse neurodegeneration. Rather, for each particular disease, medications may be available that reduce symptom burden and improve quality of life. Ongoing research efforts focus on the similarities between neurodegenerative these diseases in the hope of 1 day finding a cure. In this manuscript, notable contributions of women to neurodegeneration research are highlighted. These contributions are vast and include discoveries about neuronal pathways, proteins, vascular effects on neurodegeneration, nerve growth factor, and novel imaging techniques. In total, 12 female neuroscientists are celebrated below, namely: Rita Levi-Montalcini, Valina L. Dawson, Eva-Maria Mandelkow, Tara Spires-Jones, Maria Grazia Spillantini, Miia K. Kivipelto, Vivian Tabar, Marian Diamond, Elizabeth Roboz-Einstein, Cécile Vogt-Mugnier, Patricia Goldman-Rakic, and Anita Harding. A timeline of select key advancements in neurodegeneration research is provided below (see Figure 1).

### Rita Levi-Montalcini

Rita Levi-Montalcini was born on April 22, 1909 in Turin, Italy, and went on to make pivotal contributions to our understanding of neuroscience (Aloe, 2011). In 1947, Levi-Montalcini went to Washington University, in St. Louis, Missouri, to study nerve tissue growth in chick embryos in Viktor Hamburger's laboratory (Zeliadt, 2013). Hamburger and Levi-Montalcini discovered what they called nerve growth factor (NGF) (Aloe, 2011). Their discovery of NGF earned them the Nobel Prize in Medicine in 1986. The historic discovery of NGF has allowed scientists to study neural growth, and enhanced understanding of neurodegenerative diseases such as AD (Scott and Crutcher, 1994), PD (Mogi et al., 1999) and MS (Laudiero et al., 1992).

### Valina L. Dawson

Valina L. Dawson, professor of Neurology at Johns Hopkins Medical School, researches cell signaling pathways that lead to neuronal death (Zhang et al., 2017). She also studies EIF4G1 and LRRK2, two mutations associated with familial Parkinson's disease (Martin et al., 2014). Dawson uses animal models (Lee et al., 2012) as well as post-mortem brains (Martin et al., 2010) to explore these signaling pathways. Using an animal model, Dawson characterized a pathway of Parkinson's disease called Parthanatos (Fatokun et al., 2014). Dawson's genetic work led to insights into the complicated origins and causes of neurodegenerative diseases such as PD. Dawson has received over 60 awards and honors in her career, including Thomson Reuters: The World's Most Influential Minds, 2014 and the Javits Neuroscience Investigator Award, 2014.

### Eva-Maria Mandelkow

Eva-Maria Mandelkow is a neuroscientist who began studying tau (a protein that stabilizes axonal microtubules) in the context of AD in 1989. Mandelkow's research demonstrated that tau is one of the best indicators of the presence of AD (Mandelkow and Mandelkow, 1998), paving the way for more extensive study of this protein. Mandelkow's research involves studying the spread of tau pathology (Mudher et al., 2017) and tau's role in brain development (Sapir et al., 2012).

### Tara Spires-Jones

Tara Spires-Jones is a professor at the University of Edinburgh. Like Dawson, Jones studies mechanisms of AD and other neurodegenerative diseases. Jones' found that amyloid beta and tau proteins contribute to neurodegeneration and that lowering the levels of these proteins can “prevent and reverse phenotypes in model systems” (Spires-Jones and Hyman, 2014). Jones has also developed high-resolution imaging techniques to view post-mortem brains; her technique has provided evidence for the accumulation of amyloid and tau proteins in brains with neurodegenerative diseases (Meyer-Luehmann et al., 2008; Henstridge et al., 2015).

### Maria Grazia Spillantini

Maria Grazia Spillantini is a molecular neurologist who specializes in neurodegenerative disease mechanisms and treatment. Like Dawson and Jones, Spilantini also studies the mechanisms underlying neurodegenerative diseases. Spillantini specifically studies tau and Lewy bodies, which are abnormal proteins which aggregate within the brains in people with PD and other disorders associated with Lewy bodies (Spillantini et al., 1997a,b). Spillantini is famous for discovering the protein alpha-synuclein which is a very important component in Lewy bodies (Spillantini et al., 1997a). Further work revealed that a specific mutation in the gene encoding alpha-synuclein is associated with certain types of PD (Bennett, 2005; Nussbaum, 2017).

### Miia K. Kivipelto

Miia K. Kivipelto is a Finnish neuroscientist and professor, whose research has shown that there is a relationship between dementia and vascular risk factors, such as high blood pressure and elevated blood lipids. From 2009 to 2011, Kivipelto led a clinical trial called the Finnish Geriatric Intervention Study to Prevent Cognitive Impairment and Disability (FINGER) (Kivipelto et al., 2013). This study found that a healthy diet, exercise, and cognitive training may reduce loss of cognitive function in people who are at a high risk of dementia. Kivipelto is currently running the worldwide FINGER initiative, which aims to replicate the results in a global sample.

### Vivian Tabar

Vivian Tabar is an American neurosurgeon and researcher. Tabar has been recently approved to conduct a clinical trial which tests the ability of stem cells to repair damaged brain cells in people with PD (NCT04802733) (Phase 1). Currently, the main treatment for PD is the drug L-DOPA which was first used about 60 years ago, is non-curative, and eventually stops working. Tabar's clinical trial brings promise and hope to people suffering from PD, and represents a regenerative treatment that could be later trialed for other neurodegenerative diseases.

### Marian Diamond

Mariana Diamond was a professor at the University of California Berkeley and is considered one of the founders of modern neuroscience. She famously studied Albert Einstein's brain, and found that it contained more glial cells than the average male brain from the control group (Diamond et al., 1985). Beyond this critical work, Diamond is most lauded for her pioneering research in anatomical neuroscience. Her work highlights that neuroplasticity at the cellular level maintains neuronal connections (Dorszewska et al., 2020). Prior to her seminal research, many thought that the brain and the way it functions was determined by genetics and could not be changed (Kentner et al., 2019). Although her work was initially doubted by other scientists, it eventually gained respect and brought neuroplasticity into the limelight. Other experiments on rats demonstrated the benefits of enriched environments at any age, including a thicker cortex and enhanced learning capacity (Diamond et al., 1964).

### Elizabeth Roboz-Einstein

Elizabeth Roboz-Einstein was born in Hungary in 1904 and relocated to the United States in 1940 in response to nazi forces invading Hungary. She married Hans Einstein, the first son of Albert Einstein. In an effort to better teach her students, she studied neurochemistry. This led to her interest in neuroscience, particularly myelin, an insulating fat around nerves. She isolated the myelin basic protein (MBP) using an MS animal model (Einstein et al., 1972). This discovery allowed Roboz-Einstein and other neuroscientists to determine which specific regions of MBP were antigenic. Ultimately her work led to an improved model and progressed research on immunotherapies for MS and other demyelinating diseases (Meinl and Hohlfeld, 2002).

### Cécile Vogt-Mugnier

Vogt received her medical doctorate from Paris in 1900 and is widely renowned for her work as a neurologist. She is most famous for her discoveries regarding the neuroanatomy of the thalamus. She and her husband both played a vital role in understanding the functional anatomy of the brain and subsequent mapping of the human brain (Kreutzberg et al., 1992). She was nominated for a Nobel Prize, elected to the German Academy of Science, and was awarded the National Prize of East Germany (Kreutzberg et al., 1992).

### Patricia Goldman-Rakic

Patricia Goldman-Rakic was The Eugene Higgins Professor of Neuroscience at Yale School of Medicine. Patricia Goldman-Rakic conducted seminal research on the frontal lobe and memory (Goldman-Rakic, 1990). This work greatly enhanced our understanding of both memory and behavior in the context of neurodegenerative diseases including PD and AD (Rajkowska et al., 1998). Because of Patricia Goldman-Rakic scientists were better able to understand the basis of higher cognitive function and, ultimately, of neurodegenerative diseases such as PD and AD.

### Anita Harding

Anita Harding graduated in 1975 from the Royal Free Hospital School of Medicine and became a leading clinical scientist of her time. She is considered a pioneer of neurogenetics because she anticipated the entry of molecular genetics into neurology. Some of her major accomplishments included the first identification of the mitochondrial DNA mutation in human disease and identifying trinucleotide repeat sequences in degenerative neurological diseases such as Huntington's (Poulton and Huson, 1996).

## Conclusion

This paper celebrates the notable research contributions of 12 women, whose work enhanced scientific understanding of neurodegenerative diseases and changed the direction of neuroscience. Overall, the contributions of these and other female neuroscientists are vast and include the creation of novel imaging techniques as well as enhanced understanding of proteins, genetics, and environmental factors that contribute to brain health and neurodegenerative disorders. The work summarized in this manuscript and other contributions of women neuroscientists helped to advance neurodegenerative research, though their important contributions may not be widely known or taught in schools. This manuscript serves as a primer meant to celebrate select seminal contributions of women to neurodegeneration research; interested readers are encouraged to explore more work by these and other female neuroscientists.

## Author Contributions

CG and PG conducted literature reviews to gather research from different sources and wrote sections of the draft. CG compiled drafts to create the final manuscript and designed the figure. All authors worked to revise the draft with NO contributing significantly to outlining the paper as well as the revision process.

## Conflict of Interest

The authors declare that the research was conducted in the absence of any commercial or financial relationships that could be construed as a potential conflict of interest.

## Publisher's Note

All claims expressed in this article are solely those of the authors and do not necessarily represent those of their affiliated organizations, or those of the publisher, the editors and the reviewers. Any product that may be evaluated in this article, or claim that may be made by its manufacturer, is not guaranteed or endorsed by the publisher.
